# Challenging interpretation of germline *TP53* variants based on the experience of a national comprehensive cancer centre

**DOI:** 10.1038/s41598-023-41481-y

**Published:** 2023-08-31

**Authors:** Henriett Butz, Anikó Bozsik, Vince Grolmusz, Erika Szőcs, János Papp, Attila Patócs

**Affiliations:** 1 Department of Molecular Genetics and the National Tumour Biology Laboratory, National Institute of Oncology, Comprehensive Cancer Center, Budapest, Hungary; 2 Department of Oncology Biobank, National Institute of Oncology, Comprehensive Cancer Center, Budapest, Hungary; 3 Hereditary Tumours Research Group, Eötvös Loránd Research Network, Budapest, Hungary; 4 Department of Laboratory Medicine, Semmelweis University, Budapest, Hungary

**Keywords:** Cancer, Clinical genetics

## Abstract

*TP53* variant interpretation is still challenging, especially in patients with attenuated Li–Fraumeni syndrome (LFS). We investigated the prevalence of pathogenic/likely pathogenic (P/LP) variants and LFS disease in the Hungarian population of cancer patients. By testing 893 patients with multiplex or familial cancer, we identified and functionally characterized novel splice variants of *TP53* helping accurate variant classification. The differences among various semi-automated interpretation platforms without manual curation highlight the importance of focused interpretation as the automatic classification systems do not apply the *TP53*-specific criteria. The predicted frequency of the *TP53* P/LP variants in Hungary is 0.3 per million which most likely underestimates the real prevalence. The higher detection rate of disease-causing variants in patients with attenuated LFS phenotype compared to the control population (OR 12.5; p < 0.0001) may raise the potential benefit of the *TP53* genetic testing as part of the hereditary cancer panels of patients with multiple or familial cancer even when they do not meet Chompret criteria. Tumours developed at an earlier age in phenotypic LFS patients compared to the attenuated LFS patients which complicates genetic counselling as currently there are no different recommendations in surveillance protocols for LFS, phenotypic LFS, and attenuated LFS patients.

## Introduction

Classic Li–Fraumeni syndrome (LFS) is a high-penetrance hereditary condition predisposing to several tumour types in age-related phases^1^. In childhood, the characteristic tumour types are adrenocortical carcinoma, choroid plexus carcinoma, rhabdomyosarcoma, and medulloblastoma. During the transition from childhood to young adulthood, breast cancer, gastrointestinal, lung cancer, and different sarcomas are the most prevalent, while pancreatic and prostate cancer occur mostly during late adulthood^1^. The most characteristic tumour types are breast cancer, soft tissue sarcoma, osteosarcoma, brain tumour, and adrenocortical carcinoma which are called “core tumours”.

So far, only germline *TP53* pathogenic variants have been identified as disease-causing factors in association with this clinical condition^2^. As the clinical diagnosis is based on the presence of sarcoma in the proband and strong familial involvement (including at least two family members affected), the prevalence of the disease was estimated to be very rare without knowing the exact population frequency^3–5^. However, lately, pathogenic *TP53* variants were identified in several patients with attenuated phenotypes due to the wide application of multigene panels used by next-generation sequencing in clinical practice even in patients not fulfilling the *TP53* testing criteria^3^. Additionally, higher allele frequency of pathogenic/likely pathogenic (P/LP) *TP53* variants was also described in population databases than it would have been expected based on the disease prevalence^4,5^. This fundamentally changed our view on disease penetrance; therefore, genetic testing criteria (Chompret criteria) were widened to the identification of cases irrespective of family history (Table 1)^2,3^. Recently, the use of the following phenotype categories was suggested by Kratz et al.^3^: (1) *LFS*: the presence of a germline P/LP or mosaic *TP53* variant in a person with any cancer before age 18 years or who meets established testing criteria (Chompret A or B). (2) *attenuated LFS*: the presence of a germline P/LP or mosaic *TP53* variant in a person with any cancer who does not meet LFS genetic testing criteria and has no cancer diagnosed before age 18 years. (3) *incidental LFS*: the presence of a germline P/LP or mosaic *TP53* variant in a person/family without cancer. (4) *phenotypic LFS*: the absence of a P/LP germline or germline mosaic *TP53* variant in a person who meets classic LFS criteria or category A Chompret criteria.

**Table 1:** Chompret criteria. In our study, we investigated patients fulfilling category A Chompret criteria resembling the “classic” LFS phenotype.

Category A
Proband with a core tumour before age 46 years and ≥ 1 first- or second-degree relative with a core tumour (except a breast cancer if proband had breast cancer) before age 56 years
OR
≥ 2 tumours (not multiple breast cancers), including 2 core tumours, the first of which occurred before age 46 years
Category B
ANY of the following: Adrenocortical carcinoma, choroid plexus carcinoma, anaplastic rhabdomyosarcoma, breast cancer before age 31 years, osteosarcoma, childhood hypodiploid acute lymphoblastic leukaemia, sonic hedgehog-medulloblastoma

For variant interpretation, several sources are used in daily routine including the National Institute of Health (NIH) funded endeavours, such as ClinVar (https://www.ncbi.nlm.nih.gov/clinvar/) and Clinical Genome Resource (ClinGen, https://clinicalgenome.org/)^6^. ClinVar, is a freely accessible, public archive of reports on the relationships among human variations and phenotypes. It processes submissions reporting variants found in patient samples, assertions made regarding their clinical significance, information about the submitter, and other supporting data (https://www.ncbi.nlm.nih.gov/clinvar/intro/). It is probably the most widely used source for variant interpretation. However, due to its nature (individual submissions), discordant interpretation of variants has been often reported among ClinVar submitters and between expert reviewers^7–10^. Therefore, ClinGen performs curation of variant interpretation by variant curation expert panels, but the great majority of variants have been waiting for expert reviews. The American College of Medical Genetics and Genomics (ACMG) established guidelines for reporting and interpreting germline sequence variants in an effort to standardize clinical evaluation of genomic information which is recommended to use as a standard procedure^6,11^. Since then, different platforms (i.e. InterVar^12^, Varsome^13^ and Franklin Genoox (https://franklin.genoox.com) have been created to apply ACMG criteria and to make variant interpretation easier. Due to the particular characteristics of the *TP53* gene and the associated phenotypes (e.g., pleiotropic function and uncertain penetrance), specific criteria were recommended for variant classification^14,15^. Many P/LP variants and/or their interpretations are missing from the official NCI *TP53* database or other databases. The functional consequences of these variants are also unknown. Certain gene and patient-specific factors have to be taken into consideration during variant classification, hence, variant interpretation is still challenging in routine molecular genetic diagnostics^16^, especially in case of patients not meeting Chompret criteria.

In the Hungarian population, the prevalence of the *TP53* P/LP variants and the LFS disease are currently unknown. As a national genetic testing centre for cancer patients, we faced the abovementioned challenges. Therefore in this study our goals were (1) to assess the prevalence of *TP53* P/LP variants in Hungarian patients with cancer; (2) to compare the *TP53* detection rate in LFS, attenuated LFS, and phenotypic LFS population about which there is only scarce information, especially in a central European population; (3) to compare *TP53* P/LP rate in our cohort to non-cancer and disease-control groups; (4) to perform RNA-level characterization of novel *TP53* splice variants to aid the correct variant classification; and (5) to investigate the clinical importance of applying *TP53*-specific interpretation compared to semi-automatic web-based platforms routinely used in diagnostic genetic testing laboratories.

## Results

### TP53 variant characterization and variant interpretation

Germline genotyping of the *TP53* gene of 893 patients with multiplex or familial cancer resulted in 15 (P/LP/VUS) variants in 16 patients (NM_000546.6:c.743G > A was observed in two patients) (Table 2). Of the 15 variants, only 6 variants were present in the NCI *TP53* database^17^ (Table 3).

**Table 2:** Patient characteristics.

Sex	Age of 1st tumour (years)	Tumours in the proband (age of onset, years)	Tumours detected among family members (1st, 2nd and 3rd degree relatives)	Chompret criteria	Kratz category (3) (Phenotypic LFS/LFS/AttenuatedLFS)	*TP53* variant (according to NM_00546.6)	*TP53-*specific variant classification
M	10	Acute lymphoid leukaemia (10 years), parotid mucoepidermal carcinoma (28 years), Soft tissue leiomyosarcoma (28 years), spinocellular carcinoma (33 years)	CNS tumour, thyroid cancer	A	LFS	c.743G > A	P
F	21	Ovarian dermoid cyst (21 years), retroperitoneal leiomyosarcoma (41 years), breast Cancer (41 years), parathyroid cancer (41 years)	CNS tumour, lung cancer	A	LFS	c.589G > C	LP
M	38	Pulmonary malignant PEComa (38 years), sinonasal carcinoma (40 years), prostate Leiomyosarcoma (42 years)	Osteosarcoma, breast cancer, melanoma	A	LFS	c.97-2A > C	P
F	1	Adrenocortical carcinoma (1 years)	CNS tumour, breast cancer	A	LFS	c.460G > A	P
F	24	Breast cancer (24 years)	CNS tumour, leukaemia	A	LFS	c.536A > T	P
F	37	Dermatofibrosarcoma (37 years), renal cancer (39 years), thyroid cancer (49 years), breast Cancer (57 years)	CNS tumour, pancreas cancer, bladder cancer	A	Phenotypic LFS	–	–
F	38	Bilateral breast cancer (38, 64 years)	Adrenocortical cancer, parotid cancer, lymphoma, Colorectal cancer	A	Phenotypic LFS	–	–
F	28	Breast cancer (28 years)	Liposarcoma, breast cancer, lung cancer	A	Phenotypic LFS	–	–
F	28	Breast cancer (28 years)	CNS tumour, lung cancer, breast cancer	A	LFS	c.493C > T	LP
F	39	Acute lymphoid leukaemia (11 years), breast cancer (39 years)	Breast cancer	A	Phenotypic LFS	–	–
F	39	Breast cancer (39 years)	CNS tumour, leukaemia, colorectal cancer, breast Cancer, hepatocellular cancer, endometrial cancer	A	Phenotypic LFS	–	–
F	40	Bilateral breast cancer (61, 65 years), lymphosarcoma (71 years)	No familial data due to the holocaust	A	Phenotypic LFS	–	–
F	33	Bilateral breast cancer (33, 35 years)	Adrenocortical cancer	A	LFS	c.902delC	LP
F	35	Bilateral breast cancer (35, 51 years), melanoma (56 years)	Gastric cancer	–	No LFS	c.79C > A	VUS
F	33	Breast cancer (36 years)	Breast cancer	–	Attenuated LFS	c.323_329dup	LP
F	60	Ovarian cancer (60 years)	Hepatocellular cancer	–	Attenuated LFS	c.743G > A	P
F	58	Breast cancer (58 years)	Basalioma, breast cancer	–	Attenuated LFS	c.764_766del	LP
F	56	Bilateral breast cancer (56, 63 years)	No history of malignant disease	–	Attenuated LFS	c.614A > G	LP
F	49	Breast cancer(49 years)	Ovarian cancer, melanoma	–	No LFS	c.376-2dupA	VUS
M	61	Breast cancer (61 years)	Breast cancer, prostate cancer	–	No LFS	c.375 + 6 T > C	VUS
F	54	Breast cancer (54 years)	Ovarian cancer	–	No LFS	c.466C > T	VUS
M	64	proState cancer (64 years)	No history of malignant disease	–	Attenuated LFS	c.473G > A	P

*CNS* central nervous system, *LFS* Li–Fraumeni syndrome, *A* Chompret A category, *M* male, *F* female.

**Table 3:** Variant characteristics.

Variant name		Variant interpretation				NCBI ClinVar		GnomAD—MAF*		NCI TP53 DB	
HGVS CDNA (NM_000546.6)	HGVS protein	TP53-specific interpretation	Varsome-ACMG class	Franklin-ACMG Class	ClinGen (TP53 VCEP*)	Review status	Class	MAF total Exomes (V.2.1.1)	MAF total Genome (V.2.1.1)	TA class (transcriptional activity)	DNA LOF class
c.79C > A	p.Pro27Thr	VUS (PP3-supp; PM2-supp; PM1-supp)	VUS-LP	VUS	*n.d*	*	VUS(4), LB(1)	*n.d*	*n.d*	*n.d*	*n.d*
c.97-2A > C	p.?	P (PVS1-str; PM2-supp; PP3-mod; PS3-str; PS4-supp)	P	LP	*n.d*	*n.d*	*no data*	*n.d*	*n.d*	*n.d*	*n.d*
c.323_329dup	p.Leu111fs	LP (PVS1-str; PM2-supp)	P	P	*n.d*	**	P	*n.d*	*n.d*	*n.d*	*n.d*
c.375 + 6 T > C	p.?	VUS (PM2-supp; BS3-supp)	VUS-P	VUS	*n.d*	**	VUS	*n.d*	*n.d*	*n.d*	*n.d*
c.376-2dupA	p.?	**VUS (**PM2-supp; BS3-supp)	LP	LP	*n.d*	**	VUS	8.01E−06	*n.d*	*n.d*	*n.d*
c.460G > A	p.Gly154Ser	P (PS3-supp; PS4-supp; PS4-supp; PM1-str; PP3str)	P	VUS	*n.d*	**	VUS	3.98E−06	1,59E-04	*n.d*	*n.d*
c.466C > T	p.Arg156Cys	**VUS **(PM5-str; PM2-supp; BS3-supp)	P	LP	*n.d*	*	VUS(4), LB (3)	3.98E−06	*n.d*	partially functional	notDNE_notLOF
c.473G > A	p.Arg158His	P (PS3-str; PM5-str; PP3supp; PM2-supp)	P	P	*n.d*	**	P/LP	398E−06	*n.d*	non-functional	DNE_LOF
c.493C > T	p.Gln165Ter	LP (PS4-supp; PVS1-str; PM2-supp)	P	P	*n.d*	**	P	*n.d*	*n.d*	*NA*	notDNE_notLOF
c.536A > T	p.His179Leu	P (PP3-str; PM2-supp; PS4-supp; PM1-str)	P	LP	*n.d*	*	LP(1), VUS(1)	*n.d*	*n.d*	*n.d*	*n.d*
c.589G > C	p.Val197Leu	LP (PP3-mod; PM2-supp; PS4-mod; PM1-str)	LP	LP	*n.d*	*	VUS	*n.d*	*n.d*	*n.d*	*n.d*
c.614A > G	p.Tyr205Cys	LP (PM1-str, PM2-supp; PP3-str)	P	P	*n.d*	**	P	*n.d*	*n.d*	non-functional	DNE_LOF
c.743G > A	p.Arg248Gln	P PS3-str; PM1-str; PP3-str; PM2-supp)	P	P	P	***	P	1.19E−05	*n.d*	non-functional	DNE_LOF
c.764_766del	p.Ile255del	LP (PM1-str; PM2-supp; PS3-mod)	P	LP	*n.d*	**	LP	*n.d*	*n.d*	*n.d*	*n.d*
c.902delC	p.Pro301fs	LP (PVS1-str; PM2-supp; PS4-supp)	P	P	*n.d*	*	P	*n.d*	*n.d*	*n.d*	*n.d*

*VCEP* Variant Curator Expert Panel, *MAF* minor allele frequency, *P/LP* pathogenic/likely pathogenic, *VUS* variant of uncertain significance, *DNE* dominant negative effect, *LOF* loss-of-function. Bold letters indicate downgrading following manual curation of interpretation using gene-specific variant interpretation criteria. In the ‘TP53-specific interpretation’ column ACMG classification criteria and evidence levels are indicated in parenthesis: *str* strong, *supp* supporting, *mod* moderate.

**Figure 1:**
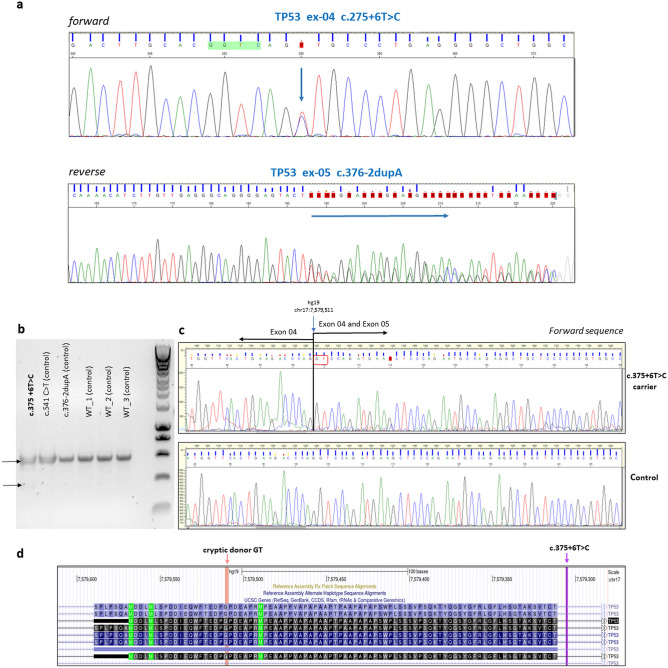
Validation of the germline *TP53* variants c.275 + 6T > C and c.376-2dupA by Sanger sequencing (**a**) and RNA level characterizations of *TP53* splice variants (**b**) Agarose gel electrophoresis of the RT-PCR products of the *TP53*:c.375 + 6T > C variant carrier and controls. A very faint, 200 bp-shorter extra band is detectable at the c.375 + 6T > C variant carrier sample (**c**) Sanger sequencing of the RT-PCR product of the *TP53*:c.375 + 6T > C variant carrier. The extremely low level of aberrant splice product of the variant carrier, generated by weakening the canonical splice donor site and applying a pre-existing cryptic exonic donor GT site (shown with red bracket, chromosome position is indicated) (**d**) Screenshot of the *TP53* exon 4 genomic region (hg 19) visualized by UCSC genome browser (https://genome-euro.ucsc.edu). The orange arrow indicates the position of the activated donor GT splice site, purple arrow marks the variant position. The measure of the aberrant splicing, although detectable, is extremely low, therefore negligible from a clinical point of view.

Among the identified *TP53* variants, 2 variants with in silico predicted splice effect were identified: NM_000546.6:c.375 + 6T > C and NM_000546.6:c.376-2dupA. Strong splice effect was predicted for both variants: In the case of NM_000546.6:c.375 + 6T > C ADA score was 0.9921, VarSEAK classified it as Class 4 and spliceAI gave a score of 0.21. The second variant, NM_000546.6:c.376-2dupA was categorized as Class 5 by VarSEAK, and spliceAI resulted in a score of 0.29. For correct variant classification, the splice effect was tested on the cDNA level. NM_000546.6:c.376-2dupA did not show any effect on splicing, while the NM_000546.6:c.375 + 6T > C variant resulted in a negligible aberrant splicing (< 10%) (Fig. 1a–d).

The variant classification was performed including these functional data as well following the specifications of the ACMG/AMP variant interpretation guidelines for germline *TP53* variants. All variants were submitted to the LOVD database.

### TP53 variant detection rate and differences in interpretation in patients with Li–Fraumeni spectrum

**Figure 2:**
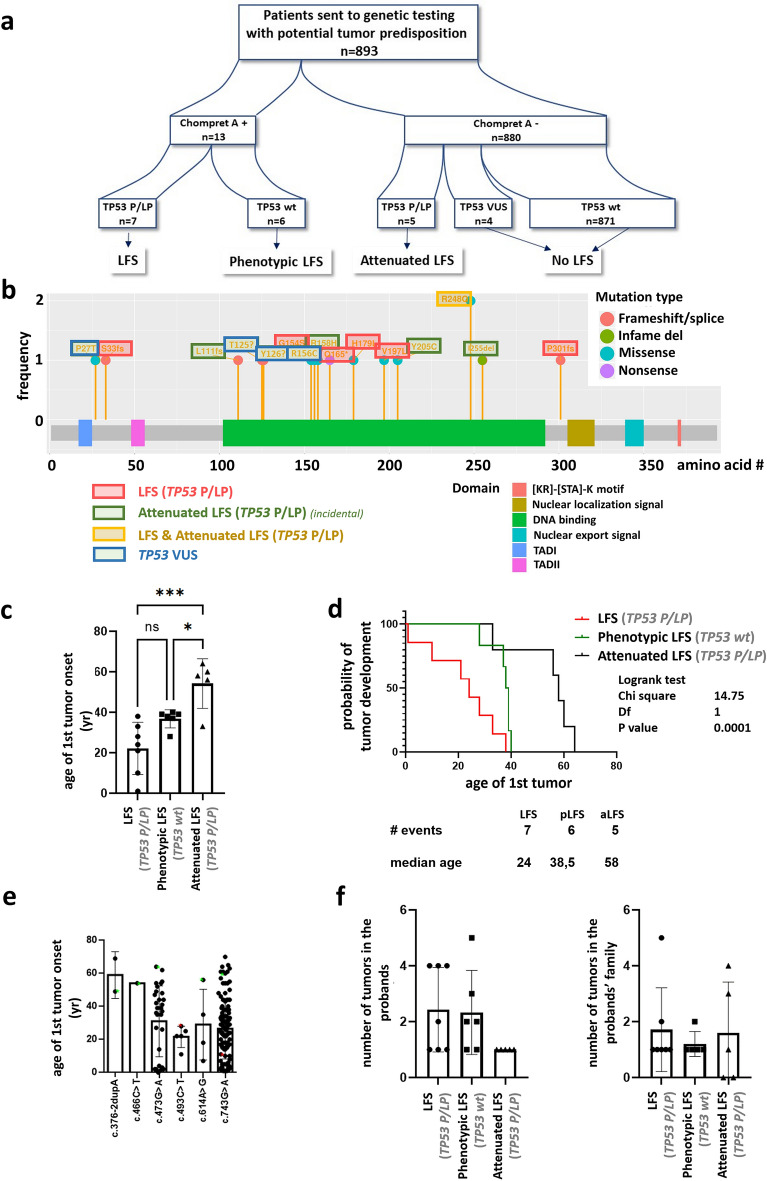
(**a**) Patient cohort and genetic testing outcome. (**b**) Localizations of the identified variants in the *TP53* gene. (**c**,**d**) Age and probability of first tumour onset in LFS, phenotypic LFS, and attenuated LFS groups. (**e**) Age of first tumour onset according to variants found in NCI *TP53* database. Green stars-attenuated LFS; red stars-LFS patients. (**f**) Number of tumours in the proband and probands’ families in LFS, phenotypic and attenuated LFS groups.

Of the 893 patients, 13 met Chompret A criteria. Among them, *TP53* P/LP variants were identified in 7 cases (7/13, 54%), while 6 patients were classified as phenotypic LFS with wild-type *TP53* gene (Table 2, Fig. 2a). None of these patients met classic LFS criteria.

Among 880 patients who did not meet Chompret A criteria (Table 4), *TP53* variants were identified in 9 cases: 5 P/LP (5/880, 0.5%) and 4 VUS. The difference in detection rate between patients meeting and not meeting Chompret A criteria indicates the good applicability for the indication of the genetic test (p < 0.0001). Two control groups were investigated to compare this data to the allele frequencies. As *population control* data, the Genome Aggregation Database (gnomAD V.2.1.1) was used applying the European non-Finnish non-cancer population (n = 59,095)^18^. As a *disease control group* clinical data of Li-Fraumeni patients carrying germline *TP53* variants were obtained from the US National Cancer Institute (NCI) *TP53* Database (a successor of the International Agency for Research on Cancer *TP53* database)^17^. This database contained data from 4455 patients with germline *TP53* variants, of these 213 patients carried the variants we found in our investigated population. In population controls, *TP53* P/LP variants were identified in 27 of the 59,095 cases (0.05%) (Supplementary Table S1), therefore the odds to detect *TP53* P/LP variant in cancer patients with multiple tumours or patients having multiplex tumours in the family but not fulfilling Chormpet A criteria was 12.5 (OR 12.5; 95% CI 4.803–32.54; p < 0.0001).

**Table 4:** Characteristics of patients not fulfilling Chompret A criteria.

Gender	
Female (n)	743
Male (n)	137
Total (n)	880
Age	
Average (y) ± standard deviation (y)	48 ± 13
Minimum (y)–maximum (y)	1–80
Total (n)	880
Disease distribution based on referral diagnosis	
HBOC syndrome-associated tumour types (breast, ovarian, pancreatic, prostate cancer)	621
Lynch syndrome-associated tumour types (colorectal, endometrial, ovarian cancer) and colon polyposis	122
Endocrine-related tumour types (e.g., thyroid, adrenal, pituitary, neuroendocrine tumours, etc.)	101
Other (e.g., melanoma, liver cancer, meningioma, melanoma, etc.)	36
Total (n)	880

*HBOC* hereditary breast and ovarian cancer syndrome, *n* number, *y* years.

By comparing literature resources regarding *TP53* variant interpretation we found remarkable differences according to the often-used web resources. Namely, 13% (2/15), 20% (3/15), and 27% (4/15) of the variants referred by Varsome, Franklin, and ClinVar respectively, were misclassified (VUS instead of P/LP or vice versa) without manual curation compared to the gene-specific interpretation results (Table 3).

### Genotype–phenotype correlations

LFS and attenuated phenotypes were not associated with variants’ localization in the *TP53* gene, as the identified genetic alterations were located throughout the whole gene irrespective of the phenotype category (Fig. 2b).

However, the age of the first tumour onset was younger in patients with LFS compared to ones having an attenuated form of the disease. The probability of tumour development and the average age of onset of the first tumour in phenotypic LFS patients (*TP53* wild-type genotype) was between LFS and attenuated LFS patients (harbouring *TP53* P/LP variant) (Fig. 2c,d). We did not find any association between the age of the first tumour onset and different *TP53* variants either among our cases or cases registered in the NCI *TP53* database (Fig. 2e). Phenotypes were not related to the number of tumours either in the probands or in the family of the probands (Fig. 2f). The lack of genotype–phenotype associations is additionally strengthened by the identification of the same pathogenic variant (NM_000546.6:c.743G > A) in a LFS and an attenuated LFS patient.

Among individuals fulfilling Chompret A criteria, there were no differences in multiple tumours occurrence, familial appearance, rare tumour type, or early-onset breast tumour development between LFS (*TP53* P/LP carriers) and phenotypic LFS patients (*TP53* wild-type genotype). We found the same relations when all *TP53* P/LP carriers (LFS together attenuated LFS group) were compared to *TP53* wild-type individuals (phenotypic LFS group).

## Discussion

Altogether we identified 12 different P/LP variants in our cancer patient cohort. In two cases functional in vitro characterization resulted in downgrading of classification category. Indeed, despite the strong effect indicated by splice predictors for variants NM_000546.6:c.375 + 6T > C and NM_000546.6:c.376-2dupA, this could not be corroborated by in vitro functional assays, indicating that in vitro testing of the splice effect is an important part of accurate variant interpretation.

Based on literature data, the P/LP *TP53* variant detection rate was reported as 17% (82/474^19^), 21% (22/105^20^), 29% (67/232^21^), or 35% (69/195^22^) in patients fulfilling Chompret criteria. In our patient cohort, this was higher (54%) which could be explained by the patient selection criteria as in our study only individuals fulfilling the Chompret A category were included (patients fulfilling Chomrpet B criteria were omitted due to the low detection rate). The prevalence of LFS in Hungary is unknown. In 4 years, we identified 13 probands fulfilling the Chompret A criteria. Our center as a national reference center in Hungary performs germline *TP53* testing. Therefore, the 13 cases of LFS syndrome identified between 2018 and 2022 in Hungary (having approx. 10 million inhabitants) estimate the incidence as 0.32 per million. This data most likely underestimates the real prevalence, however, the 5 cases out of the 880 unselected cancer patients with P/LP *TP53* variants identified during 1 year would mean 0.5% prevalence. Further epidemiological studies are warranted to determine the real prevalence of LFS in Hungary. Our P/LP *TP53* variant frequency data observed in cancer patients are in line with earlier published data. The 0.5% incidence rate detected using multigene panel testing strategy in our consecutive cancer patients is similar to those reported by Rana et al. They detected 126 *TP53* mutant cases out of 40,885 tested cancer patients (0.3%)^23^.

We found a higher P/LP *TP53* variant detection rate in cancer patients not fulfilling Chompret criteria (attenuated LFS phenotype) compared to the non-cancer GnomAD control population (OR 12.5; p < 0.0001). This raises the potential benefit of the *TP53* genetic testing as part of the hereditary cancer panel of patients with multiple or familial cancer even when they do not meet Chompret criteria. Identifying most of the cases with the germline P/LP *TP53* variant allows in these families, the possibility for genetic counselling followed by genetic testing for asymptomatic first-degree relatives. Then, in mutation carriers the most adequate clinical interventions can be started, hence the best prevention can be achieved.

The identified differences among different semi-automated interpretation platforms with and without manual curation highlight the importance of focused interpretation especially, since the automatic classification systems apply “classic” ACMG classification criteria^11^ instead of *TP53*-specific ACMG criteria^14^. In addition, patient and family-specific data together with in vitro functional data should be included for precise variant interpretation^14–16^.

In our cohort, we did not observe genotype–phenotype correlation except that, expectedly, the first tumour onset was earlier in LFS patients compared to attenuated LFS individuals. Interestingly, tumours developed at an earlier age in phenotypic LFS patients (*TP53* wild-type genotype) compared to attenuated LFS patients (harbouring *TP53* P/LP variant). Fortuno et al. observed similar findings, patients carrying truncating and hotspot variants experienced an earlier cancer diagnosis and might be more prone to present with LFS malignancies compared to carriers of other *TP53* variant types^24^. However, the authors concluded that the observed differences were minor, and current evidence was insufficient to consider genotype–phenotype associations to assist with the clinical management of *TP53* carriers^24^. These observations complicate genetic counselling as currently there are no distinguished recommendations in surveillance protocols for LFS, phenotypic LFS, and attenuated LFS patients^2,3,18^. However, our findings raised the possibility of delayed starting age for surveillance protocols in attenuated LFS cases that would relieve the burden of the exhausting childhood surveillance program in those patients and their families. Naturally, this should be further validated on larger cohorts, consensual international findings, and longer follow-up of cancer patients with attenuated LFS phenotype.

Study limitations should be declared, namely, the small sample number limits the assessment of genotype–phenotype associations. Our centre is the only Comprehensive Cancer Centre in Hungary, performing germline testing for cancer patients and it is assumed that all cases with a suspicion of having LFS might be referred to our centre, but we feel that not all cases have been referred during the tested period. Further efforts (including further development of the nationwide cancer registry and education of members of regional cancer centres) are needed for better estimates of the real prevalence of rare cancer types in Hungary. Despite that all results with P/LP variants and VUSs were validated on a second independently extracted DNA sample using Sanger sequencing (showing 100% concordant results with the NGS method), we cannot exclude entirely the possibilities of age- and/or therapy-related clonal haematopoiesis.

## Materials and methods

### Subjects

*TP53* gene was investigated in 880 consecutive oncology patients referred for molecular genetic testing at our national centres (Department of Laboratory Medicine, Semmelweis University and Department of Molecular Genetics, National Institute of Oncology) between 2021 and 2022. This cohort consisted of patients with potential hereditary tumour predisposition. Their genetic analysis was performed by a multigene panel within routine clinical genetic care.

Targeted *TP53* analysis was performed in 13 patients with multiple tumours or multiplex tumours in the family where clinicians suspected Li-Fraumeni syndrome (fulfilling Chompret A criteria) between 2018 and 2022 (Table 2) according to Kratz et al.^3^. In our study, we investigated patients fulfilling category A criteria resembling the “classic” LFS phenotype and patients fulfilling Chomrpet B criteria were omitted due to the low detection rate. The phenotypic classification was applied following the recommendation by Kratz et al.^3^.

According to Hungarian legal and ethical regulations, germline genetic analysis was performed following genetic counselling. Each patient gave informed consent to the genetic test based on the approval of the Scientific and Research Committee of the Medical Research Council of the Ministry of Health, Hungary (ETT-TUKEB 53720-4/2019/EÜIG, ETT-TUKEB 4457/2012/EKU).

All research was performed in accordance with the Declaration of Helsinki and informed consent was obtained from all participants.

To compare allele frequencies, two control groups were investigated. *Population control* data from Genome Aggregation Database (gnomAD V.2.1.1) was used applying the European non-Finnish non-cancer population (n = 59,095)^18^. As a *disease control group* clinical data of Li–Fraumeni patients carrying germline *TP53* variants were obtained from the US National Cancer Institute (NCI) *TP53* Database (a successor of the International Agency for Research on Cancer *TP53* database)^17^ containing data from 4455 patients with germline *TP53* variants.

### Genetic analysis

DNA extraction from peripheral blood was performed using the Gentra Puregene Blood Kit (#158389, Qiagen, Hilden, Germany)^25^. *TP53* genotyping and copy number analysis were carried out by conventional Sanger sequencing and MLPA (P056-D1, MRC-Holland, Amsterdam, The Netherlands) as previously described^25^, or next-generation sequencing (NGS) using the TruSight Hereditary Cancer Panel (#20029551, Illumina, San Diego, CA, USA). Sequencing was run on an Illumina MiSeq instrument with MiSeq Reagent Kit v3 (600 cycles) (#MS-102-2002, Illumina, San Diego, CA, USA).

Data analysis was run on Illumina BaseSpace Dragen Germline pipeline v2.1, and variants were called between 20 and 70% variant allele frequency (VAF), and validated by conventional Sanger sequencing. All investigated VAF ratios were close to 50% except in one case, where VAF was 22%. In this case, unfortunately, family members did not accept genetic testing to assess inheritance, and other tissues were not available to investigate mosaicism. However, in every case—including the one with VAF 22%—all P/LP variants and VUSs were validated on a second independently extracted DNA sample using Sanger sequencing and showed 100% concordant results with the NGS method.

### RNA level characterizations of *TP53* splice variants

The effects of the *TP53* variants on splicing were predicted in silico by ADA score, VarSEAK and spliceAI algorithms. ADA predicts within splicing consensus regions (https://help.genoox.com/en/articles/4341424-prediction-tools-and-score-range). Their potential of altering splicing by using ensemble score computed using the AdaBoost algorithm on the outputs of several other prediction tools. ADA score can range from 0 to 1, and it can be interpreted as the probability of the variant being splice-altering. VarSEAK categorizes variants as Class 4 with likely splicing effect and Class 5 with splicing effect. SpliceAI, using a 10 kb window, predicts altered splicing by scores ≥ 0.2, and it does not predict alterations when scores are ≤ 0.1.

For testing the splice effect experimentally, RNA analysis was performed as previously reported^25,26^. Briefly, RNA extraction from blood samples drawn to Tempus Blood RNA Tubes was performed by using the TempusSpin RNA Isolation Kit (Thermo Fisher Scientific, Waltham, MA, USA). RNAs then were reverse transcribed by Protoscript II First Strand Synthesis Kit (New England Biolabs, MA, USA) using random hexamer primers. cDNA was PCR-amplified, and RT-PCR products were subjected to agarose gel electrophoresis and Sanger sequencing (10,11). Amplification of the NM_000546.6:c.375 + 6T > C and NM_000546.6:c.376-2dupA variant was performed with primers 5′-AGGAAACATTTTCAGACCTATGGA-3′ and 5-CTGTCATCCAAATACTCCACACG-3′ from the cDNAs. Control samples include other *TP53* variant carriers (c.541 C > T and c.376-2dupA) as well as *TP53* variant non-carriers (WT_1-3).

### Data analysis and variant classification

NGS data were analyzed by the Illumina Dragen Germline pipeline (Dragen version 4.0.3, Illumina) where both sequence variants and copy number alterations were assessed. GRCh37 genome build and NM_000546.5 (MANE Select transcript) were used as reference sequence. Variants were reclassified following the guidelines of the ClinGen *TP53* Expert Panel specifications^14,15^. *TP53* variants were also cross-checked in the NCI *TP53* database, NCBI ClinVar (https://www.ncbi.nlm.nih.gov/clinvar/), Clinical Genome Resource (https://clinicalgenome.org/), Varsome (https://varsome.com/) and Franklin (https://franklin.genoox.com/clinical-db/home) databases. Variant interpretation and cross-referencing in different databases were done between 2 and 21 December 2022, therefore the abovementioned database data are presented accordingly.

### Statistical analyses

Statistics were carried out by GraphPad Prism 9. Depending on sample size, two-sided Fisher’s exact test or Chi-square test with Yates correction were used to compare allele frequencies between cases and population controls and to calculate ORs and 95% CIs. Analysis of variance with Tukey's multiple comparisons test was applied to compare different groups. Comparison of the probabilities of tumour development according to the age of the first tumour was calculated by log-rank test. Results were considered statistically significant when p < 0.05.

### Ethics approval

The study was approved by the Scientific and Research Committee of the Medical Research Council of the Ministry of Health, Hungary (ETT-TUKEB 53720-4/2019/EÜIG, ETT-TUKEB 4457/2012/EKU).

### Consent to participate

Informed consent was obtained from all individual participants included in the study.

### Supplementary Information

Supplementary Figure 1.

Supplementary Information.

Supplementary Table S1.

## Data Availability

The datasets generated during and/or analysed during the current study are presented in the current manuscript and are available from the corresponding author on reasonable request. All detected variants were uploaded to LOVD repository database (Accession IDs: 434602, 434604, 434611, 434612, 434613, 434614, 434615, 434632, 434633, 434634, 434635, 434636, 434637, 434641, 434644), and can be accessed at https://databases.lovd.nl/shared/screenings#order=id%2CASC&search_owned_by_=bozsik&page_size=100&page=1.
