# Supplementary figures and images for: Challenging interpretation of germline TP53 variants based on the experience of a national comprehensive cancer centre
Source: Sci Rep. 2023 Aug 31;13:14259. doi: 10.1038/s41598-023-41481-y (PMC10471726; doi:10.1038/s41598-023-41481-y)

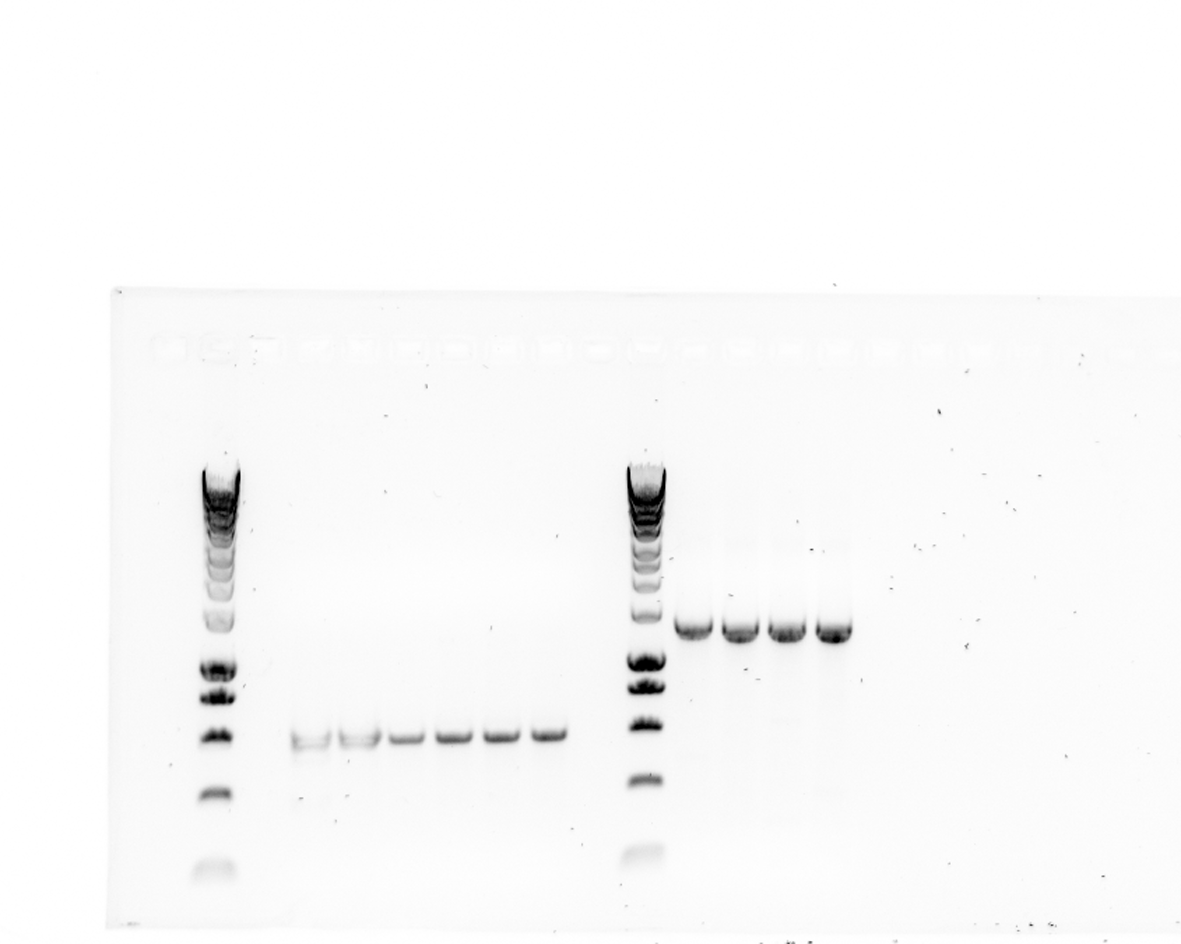

Supplement: Supplementary file 1 — Supplementary Figure 1. [file 41598_2023_41481_MOESM1_ESM.tif]
