# Supplementary material for: Challenging interpretation of germline TP53 variants based on the experience of a national comprehensive cancer centre
Source: Sci Rep. 2023 Aug 31;13:14259. doi: 10.1038/s41598-023-41481-y (PMC10471726; doi:10.1038/s41598-023-41481-y)
Supplement: Supplementary file 2 — Supplementary Information. [file 41598_2023_41481_MOESM2_ESM.docx]

Original agarose gel photo
